# Intrinsic hepatocyte dedifferentiation is accompanied by upregulation of mesenchymal markers, protein sialylation and core alpha 1,6 linked fucosylation

**DOI:** 10.1038/srep27965

**Published:** 2016-06-22

**Authors:** Anand Mehta, Mary Ann Comunale, Siddhartha Rawat, Jessica C. Casciano, Jason Lamontagne, Harmin Herrera, Aarti Ramanathan, Lucy Betesh, Mengjun Wang, Pamela Norton, Laura F. Steel, Michael J. Bouchard

**Affiliations:** 1 Drexel University College of Medicine, Department of Microbiology and Immunology, 245 N. 15th Street, Philadelphia, PA 19102, USA; 2 Graduate School of Biomedical Sciences and Professional Studies, Drexel University College of Medicine, Molecular and Cellular Biology and Genetics Graduate Program, 245 North 15th Street, Philadelphia, PA 19102, USA; 3 Graduate School of Biomedical Sciences and Professional Studies, Drexel University College of Medicine, Microbiology and Immunology Graduate Program, 2900 Queen Lane, Philadelphia, PA 19129, USA; 4 Drexel University College of Medicine, Department of Microbiology and Immunology, Institute for Molecular Medicine and Infectious Disease, 245 North 15th Street, Philadelphia, PA 19102, USA; 5 Drexel University College of Medicine, Department of Biochemistry and Molecular Biology, 245 N. 15th Street, Philadelphia, PA 19102, USA.

## Abstract

Alterations in N-linked glycosylation have long been associated with cancer but for the most part, the reasons why have remained poorly understood. Here we show that increased core fucosylation is associated with de-differentiation of primary hepatocytes and with the appearance of markers indicative of a transition of cells from an epithelial to a mesenchymal state. This increase in core fucosylation was associated with increased levels of two enzymes involved in α-1,6 linked fucosylation, GDP-mannose 4, 6-dehydratase (*Gmds*) and to a lesser extent fucosyltransferase 8 (*Fut8*). In addition, the activation of cancer-associated cellular signaling pathways in primary rat hepatocytes can increase core fucosylation and induce additional glycoform alterations on hepatocyte proteins. Specifically, we show that increased levels of protein sialylation and α-1,6-linked core fucosylation are observed following activation of the β-catenin pathway. Activation of the Akt signaling pathway or induction of hypoxia also results in increased levels of fucosylation and sialylation. We believe that this knowledge will help in the better understanding of the genetic factors associated with altered glycosylation and may allow for the development of more clinically relevant biomarkers.

Despite advances in medical technology, the 5-year survival rate for hepatocellular carcinoma (HCC) is only 8–13%, likely due to the fact that the majority of patients with HCC are diagnosed at advanced stages^1^. HCC is one of the most common solid malignancies worldwide, and the incidence in the United States is increasing^2^. In this setting of a significant increase in the number of patients with HCC, early detection and treatment are vital to improve the otherwise dismal outcome of this disease^3^,^4^.

The urgent need for biomarkers for the early detection of HCC is widely recognized, and many strategies are being pursued to achieve this goal. In our previous work, we identified increased levels of core and outer-arm fucosylation of a large number of serum and liver tissue proteins as being associated with HCC^5^,^6^,^7^,^8^ and similar increases in fucosylation have been seen in other cancers^9^,^10^,^11^,^12^,^13^,^14^,^15^,^16^,^17^.

In an effort to further understand why these changes in glycosylation occur we have used cultured primary rat hepatocytes (PRH) to study the consequence of an epithelial to mesenchymal transition (EMT) on N-linked glycosylation. In addition, we provide evidence that activation of specific cancer-associated pathways associated with an EMT in PRH can mimic alterations in glycosylation seen in human hepatocellular tumors. Further, we show that similar changes in glycosylation occur with the degree of de-differentiation of both PRH and human tumors. These findings suggest a strategy for the study of HCC that has arisen as a result of deregulation of known cell signaling pathways, and, that increased core fucosylation can be an indicator of the de-differentiation of hepatocytes observed in HCC.

## Results

### Alteration in glycosylation of primary rat hepatocytes is associated with de-differentiation and induction of an epithelial to mesenchymal transition

**Figure 1:**
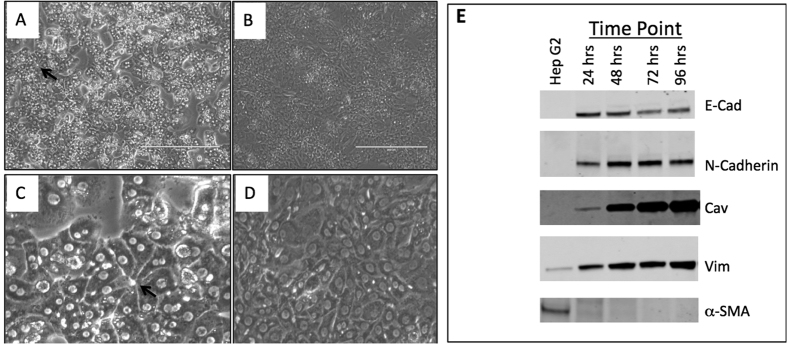
Primary rat hepatocytes undergo an EMT as a function of time in culture. 10X phase contrast microscopy of PRH at 24 (**A**) or 96 hours (**B**) post culture. (**C,D**) are magnified images highlighting either typical hepatic architecture with di-nucleated cells and bile canaliculi at the early time point (**C**) or where this is lost at the later time point (**D**). (**E**) Protein level of E-cadherin (E-Cad), N-cadherin (N-Cad), caveolin-1 (Cav), vimentin (Vim), and alpha smooth muscle actin (α-SMA) a function of time in culture. Proteins were detected by immunoblot analysis at indicated times post-culture; lane M is the marker lane. Actin is shown as a loading control. For Fig. 1E, Hep G2 lysates were used as a control as the vimentin and SMA antibodies cross react with human proteins. Figure 1E contains cropped images highlighting the change in E-cadherin (E-Cad), N-cadherin (N-Cad), caveolin-1 (Cav), vimentin (Vim), and alpha smooth muscle actin (α-SMA) as a function of time. Analysis of E-Cad, Cav and Vim were performed on the same membrane and thus under the same experimental conditions.

Our laboratory has worked extensively with primary rat hepatocytes (PRH) and used this system to study many aspects of hepatitis B virus (HBV) replication^18^,^19^,^20^,^21^,^22^. PRH, similar to murine hepatocytes, will eventually de-differentiate when kept in culture for extended periods^23^,^24^,^25^. This can be seen through the loss of normal hepatocyte cell structure (Fig. 1A–D), loss of E-cadherin (Fig. 1E) and induction of mesenchymal markers such as N-cadherin, vimentin, and caveolin-1 (Fig. 1E). Figure 1 shows a 10X phase contrast image of PRH at the different time points and highlights the loss of normal hepatic architecture. That is, as indicated in the arrow in Fig. 1A,C, at the 24 hour time points, PRH maintain hepatic architecture and bile canaliculi can be imaged by phase contrast microscopy. In contrast, this morphology is gone by 96 hours and the cells now resemble fibroblasts (Fig. 1B,D). Figure 1E shows the expression of the mesenchymal markers N-cadherin, vimentin, and caveolin-1 as a function of time in culture along with the epithelial marker E-cadherin. As Fig. 1E shows, consistent with the microscopy and what others have seen in murine primary hepatocytes, PRH in culture will undergo an EMT and de-differentiate^23^. Importantly, as seen in Fig. 1E, no expression of alpha smooth muscle actin (α-SMA) was observed in this culture, at any time point. This finding indicates that no fibroblasts were observed in the culture during isolation and that no stellate cells were present in the culture that could have potentially differentiated to myofibroblasts over the period of the study^26^. Additionally, we have examined the proteins associated with these cells through proteomics at pre and post EMT time points. These data are shown in Supplementary Figure S1, Supplementary Table 1 and highlight the changes that occur in these cells. Changes in proteins like fibronectin, N-cadherin, plasminogen, and hemopexin reflect the cellular alteration that has occurred in these cells and indicate that these hepatocytes have undergone an EMT and have de-differentiated from their normal hepatocyte state^27^,^28^,^29^. A complete list of proteins associated in the PRH at time 24 hours and time 96 hours by proteomics is found in Supplemental Table 1. Again, consistent with the data presented in Fig. 1E, proteins such as α-SMA, connexin 43, Lhx2 (encoding LIM homeobox 2), or Desmin, which are known markers of stellate cells and myofibroblasts^26^, were not observed at any time point, suggesting the lack of these cell types in the initial culture or their expansion at the later time point. It is also noted that these were also not seen in an independent RNA-Seq analysis of the PRH^22^.

**Figure 2:**
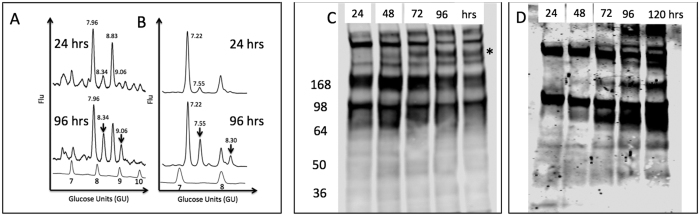
EMT is associated with alteration in glycosylation in PRH. (**A**) Normal phase HPLC chromatogram of PRH associated N-linked glycan at 24 and 96 hours post-culture. (**B**) The same samples after treatment with *Arthrobacter ureafaciens sialidase* to remove sialic acid. The core fucosylated bi-antennary and tri-antennary glycans that are increased at the 96 hour time point have glucose unit (GU) values of 7.55 and 8.30 and are indicated with an arrow in the 96 hour time point. For panels A and B glucose values are provided for the major peaks and the GU ladder is provided along the X-axis. Y-axis for these panels represents the fluorescent intensity of glycan. (**C,D**) Lectin blot analysis of independently isolated PRH at 24, 48, 72, 96 or 120 hours post culture. 10 μg of total cellular protein was resolved by SDS-PAGE and sialylated protein (**C**) or fucosylated protein (**D**) detected by lectin blotting using the *Sambucus nigra* lectin (SNA), which binds to α-2,6 and to a lesser degree to α-2,3 linked sialic acid or the fucose binding lectin N224Q rAAL lectin (see Methods). In panel C, specific bands are shown to be increased are indicated by the asterisk.

Next, we sought to determine whether changes in N-linked glycosylation could be observed in these cells as a function of time in culture and cell differentiation. Thus we performed N-linked glycan analysis of the cell associated proteins as a function of time in culture. The N-linked glycans in PRH were analyzed at time 24 or 96 hours post culture (Fig. 2A). In this method, N-linked glycans are enzymatically released from proteins by treatment with PNGase F, labeled with a fluorescent dye, and resolved via ultra high performance liquid chromatography (HPLC) into discreet chromatographic peaks as has been done previously^30^,^31^,^32^. The chromatographic peaks are assigned glycan identifications based upon retention times that are converted to glucose units (GU) using a glucose homopolymer standard curve and confirmed via treatment with exoglycosidases^6^,^14^,^33^,^34^,^35^,^36^,^37^,^38^,^39^,^40^,^41^. As shown in Fig. 2, altered peaks are observed as a function of time in culture and this reflects an alteration in glycosylation. Panel A shows the HPLC profiles of the samples after treatment with mannosidase and identifies the two peaks altered in the 96 hour time point to be mono- (GU 8.34) and di-sialyated (GU 9.06) core fucosylated bi-antennary glycan. Panel B shows the shift of the HPLC profile following sialidase treatment to remove all terminal sialic acid sugars. In addition to the increase in bi-antennary core fucose (GU 7.55) an increase in core-fucosylated tri-antennary glycan (GU 8.30) was also observed in the 96 hour time point.

Figure 2 includes an orthogonal conformation of these changes as performed by lectin blotting on separate samples at 24, 48, 72, 96 and 120 hours post culture. Panel C shows alterations in the sialic acid composition of the PRH glycoprotein pool using the *Sambucus nigra agglutinin* lectin (SNA), which binds to α-2,6 and to a lesser degree to α-2,3 linked sialic acid. Although SNA cannot confirm an increase in di-siaylated structures as shown in the HPLC profile, it does confirm that consistent changes in the sialic acid composition do occur over time as shown by the asterisk in Fig. 2C. Panel D shows the increased fucosylation over time using a recombinant *Aleuria aurantia* lectin with greater affinity for core fucose glycan, N224Q rAAL^42^ (see Methods). Figure 2C,D are consistent with the HPLC glycan analysis in that sialic acid composition changes and fucosylation increases in the PRH following time in culture.

**Figure 3:**
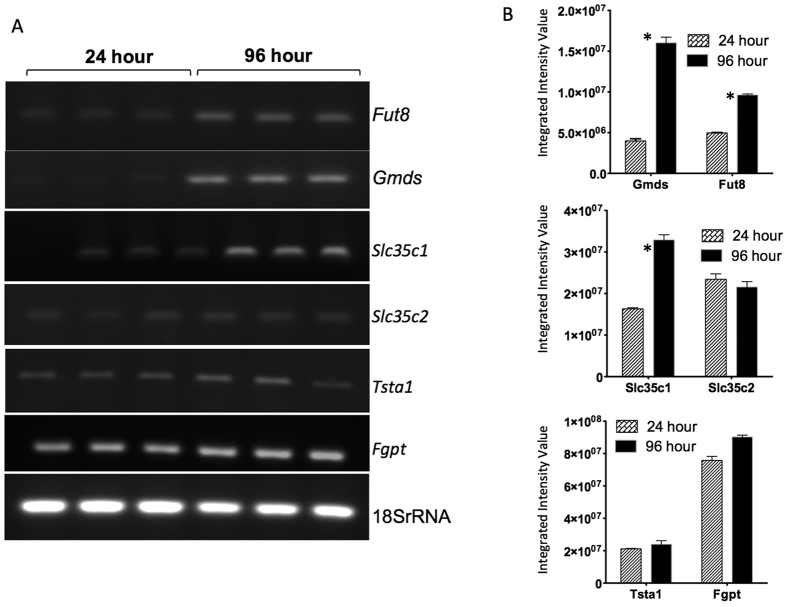
EMT is associated with alteration in key enzymes involved in fucosylation. (**A**) Expression of fucosyltransferase 8 (*Fut8*), GDP-mannose 4, 6-dehydratase (*Gmds*), GDP-fucose transporters (*Slc35c1 and Slc35c2),* GDP-4-keto-6-deoxy-mannose-3,5-epimerase-4-reductase (*Tsta1)*, and GDP- fucose pyrophosphorylase (*Fgpt*) were examined by reverse transcriptase PCR (RT-PCR) in this study. RNA analysis was performed on triplicate samples. (**B**) Quantification of the genes analyzed in panel A. The asterisks represent statistical significance (p < 0.05). For space concerns, Fig. 3A contains cropped images showing the PCR product of the different genes.

To determine whether these changes in fucosylation are accompanied by changes in the enzymes involved in the fucosylation pathway, we examined the expression level of mRNAs encoding the major enzymes involved in core fucosylation^43^. Results of reverse transcription-PCR (RT-RCR) analysis of mRNA levels in cells at 24 and 96 hours of culture are shown in Fig. 3. Consistent with the alterations in N-linked glycosylation observed by HPLC glycan sequencing and by lectin blotting, changes in many of the mRNAs associated with core fucosylation were altered as a function of time in culture. Specifically, the expression of the mRNA encoding GDP-mannose 4, 6-dehydratase (*Gmds*), the enzyme responsible for the first step of synthesizing GDP-fucose from GDP-mannose, increased from the 24 hour to 96 hour time point. Similarly, fucosyltransferase 8 (*Fut8*) mRNA showed a modest increase at the same time points. Alterations in the expression of mRNA encoding one of the GDP-fucose transporters (*Slc35c1)*, which is involved in the transport of GDP-fucose was altered in this system as well, while the related *Slc35c2* mRNA was not. The mRNA that encodes GDP-4-keto-6-deoxy-mannose-3,5-epimerase-4-reductase (*Tsta1*), the enzyme responsible for the second and third steps of the synthesis reaction, exhibited no change in expression over this time period. Similarly, no change was observed in the expression of the GDP- fucose pyrophosphorylase (*Fgpt*) mRNA. Conformation of alterations in these mRNAs was obtained through RNA-seq analysis of the entire PRH transcriptome and this work is presented in an independent publication^22^.

### Inhibition of an EMT prevents increases in core fucosylation

**Figure 4:**
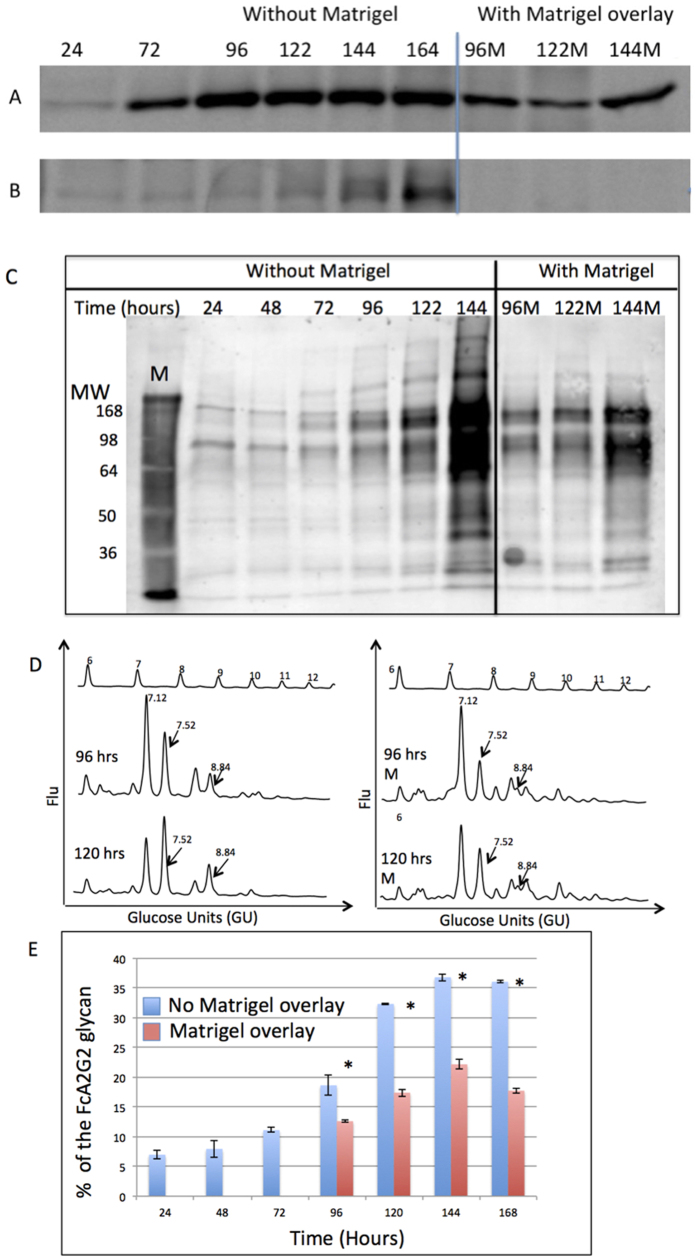
Inhibition of EMT prevents increased levels of fucosylation. PRH were grown in culture for 60 hours when the process of de-differentiation had begun. At 60 hours, Matrigel was layered on top of the PRH to inhibit the process of de-differentiation. Cells were lysed at the indicated time points and analyzed by immunoblotting to detect (**A**) Caveolin-1 or (**B**) Vimentin. (**C**) Lectin blot using N224Q rAAL to detect fucosylated proteins in lysates from cells prepared as in panels (A,B). **(D**) N-linked glycan analysis of PRH lysates after 96 and 120 hours of culture without (left) or with (right) Matrigel overlay. The arrows indicate fucosylated glycan peaks that are altered at GU values 7.52 and 8.84. For panel D, glucose values are provided for the major peaks and the GU ladder is provided along the X-axis. Y-axis for these panels represents the fluorescent intensity of glycan. (**E**) Levels of core fucosylated bi-antennary glycan at the specific times points either without or with Matrigel overlay. In Fig. 4E, the asterisks represent statistical difference (p < 0.05) in samples. For space concerns, Fig. 4A contains cropped images showing the change in Cav or Vim. Analysis of Cav and Vim were performed on the same membrane and thus under the same experimental conditions.

To further examine the relationship between core fucosylation and cellular differentiation, we used a cell culture technique to reverse the process of de-differentiation. This method utilizes a Matrigel overlay at points in time where de-differentiation has occurred to force the reversal of de-differentiation. The de-differentiation process can be monitored by measuring levels of the canonical markers of EMT (as seen in Fig. 1E). By 72–96 hours of culture, increased expression of the EMT markers caveolin-1 and vimentin were observed (Fig. 4A,B). In contrast, application of the Matrigel overlay led to reduced expression of these EMT markers, indicative of a suppression of EMT (compare the 96 to 96M and 122 and 122M lanes). Importantly, under these culture conditions, reduced levels of fucosylation are observed in the same time frame, as shown by lectin blotting using the N224Q rAAL (Fig. 4C) and by N-linked glycan analysis (Fig. 4D,E). In Fig. 4D the 96 and 120 time points without the matrigel overlay (96 and 120) show a higher level of core fucosylated bi-antennary glycan (GU 7.52) as compared to the 96 and 120 time points with the matrigel overlay (96M and 120M). Overall, these results demonstrate that reductions in fucosylation correlate with the reduction of markers of EMT.

### Increased fucosylation in poorly differentiated HCC tissue

**Figure 5:**
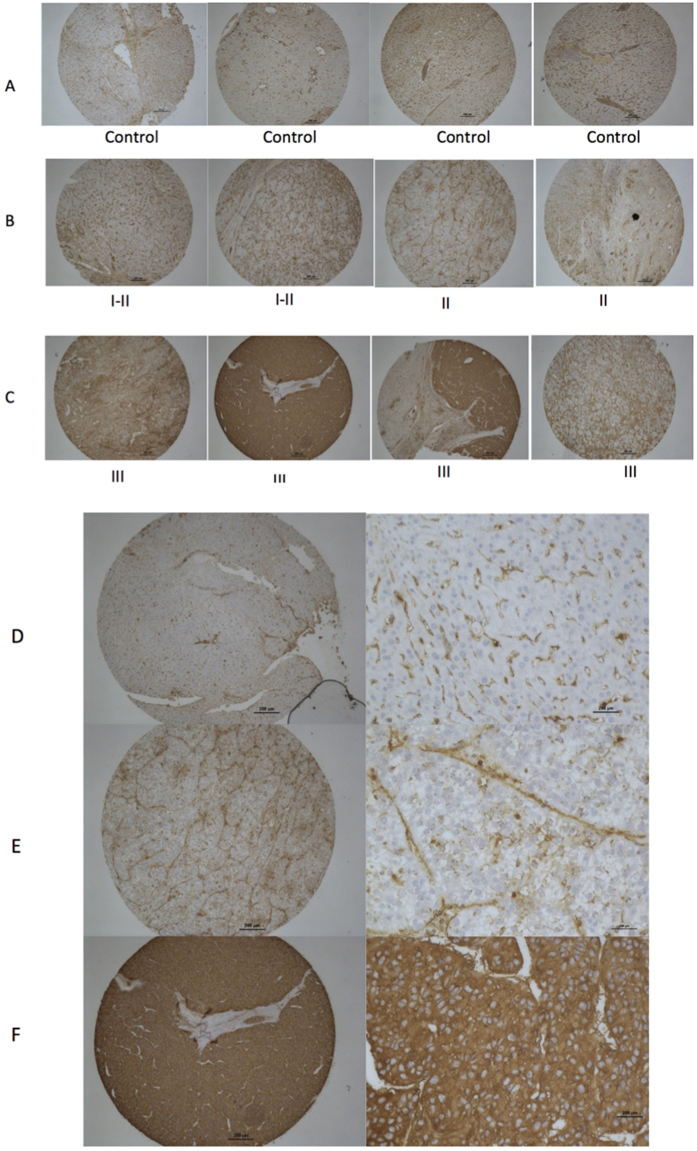
Increased fucosylation is observed with de-differentiated HCC by lectin staining with the N224Q AAL lectin. Lectin histochemistry to detect fucosylated proteins was performed using the N224Q lectin on either normal (**A,D**) or HCC tissue (**B,E**) and (**C,F**). (**A**) 4X microscopy of four tissue spots of tumor-adjacent normal tissue. (**B**) 4X microscopy of four tissue spots of Grade I-II or II HCC tissue. Grade I is defined as well-differentiated (low grade) and Grade II is defined as moderately differentiated (intermediate grade). (**C**) 4X microscopy of four tissue spots of Grade II-III or Grade III HCC tissue. Grade III is defined as poorly differentiated tissue. (**D**) Microscopy of normal, tumor-adjacent tissue. (**E**) grade I-II HCC tissue, (**F**) grade III HCC tissue showing both a 4X image and a 10X image. In all cases lectin staining was performed using N224Q rAAL.

Because increased levels of core fucosylation were associated with an EMT and de-differentiation in the PRH cell culture model, it was of interest to determine if a similar observation could be made in human HCC tissue. Thus, we examined HCC tissue microarrays (TMA) that include tissue from patients with varying grades of liver cancer. In total, we examined 16 HCC tissues with matching adjacent non-disease tissue (see Table 1). Eleven of these contained grade I-II tumor tissue (defined as well-differentiated or moderately-differentiated tissue) and 5 contained grade II-III tumor tissue (defined as poorly differentiated). The level of fucosylation was analyzed by lectin histochemistry using the N224Q rAAL that has enhanced affinity for core fucosylated glycan as opposed to other fucose linkages^42^. In our analysis, only 2 out of the 11 grade I-II tumors stained positive with the N224Q rAAL. In contrast, 4 out of 5 of the grade II-III tumors stained positive with the N224Q rAAL. Whereas hepatocytes in normal adjacent tissue do not stain with the fucose binding lectin, the hepatocytes in HCC tissue does stain, and the level of staining correlates with the tumor grade (Fig. 5). That is, while the well-differentiated tissue has little or no staining, greater levels of lectin staining are observed with the loss of cellular differentiation, with the greatest staining observed in tissue that is poorly differentiated (Table 1). (All HCC tissues that are part of the TMA are presented in Supplementary Figures S2 and S3.)

### Activation of the β-catenin signaling pathway leads to increases sialylation and fucosylation of PRH proteins

**Figure 6:**
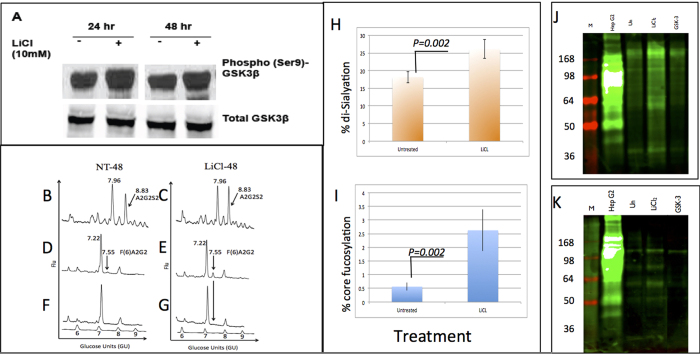
Changes in glycosylation with LiCl-induced activation of β- catenin detected by normal phase HPLC and lectin blotting. (**A**) Confirmation of LiCl effects in primary rat hepatocytes. Cells treated with LiCl for 24 or 48 hours. Phospho-GSK3β levels were analyzed by immunoblot. (**B**–**G**) Glycans were released from proteins from PRH left untreated (NT-48) or treated with LiCl for 48 hours (LiCl-48) and analyzed by normal phase HPLC as described in the text. (**B,C**) N-linked glycan profiles from untreated and LiCl treated cells. An altered glycan peak in LiCl-treated cells is indicated (arrow). (**D,E**) Samples further digested with *Arthrobacter ureafaciens* sialidase. An increased level of core fucosylation at GU7.55 in LiCl-treated cells is indicated (arrow). (**F,G**) Bovine kidney fucosidase treatment shifts this peak, indicating that it represents core fucosylation. For panels B–G, glucose values are provided for the major peaks and the GU ladder is provided along the X-axis. Y-axis for these panels represents the fluorescent intensity of glycan. (**H,I**) Quantification of the alteration in sialylation (**H**) and fucosylation (**I**) following LiCl treatment. (**J,K**) Lectin blot analysis of independently isolated PRH, left untreated or LiCl-treated for 48 hours. 10 μg of total cellular protein was resolved by SDS-PAGE and sialylated protein (**J**) or fucosylated protein (**K**) was detected by lectin blotting. Sialylated proteins were detected using the *Sambucus nigra* lectin (SNA), which binds to α-2,6 and to a lesser degree to α-2,3 linked sialic acid. (**K**) Increases in fucosylation were detected using the core fucose binding lectin N224Q rAAL (see Methods). HepG2 hepatoblastoma cells serve as a positive control (+). For J and K: M is marker lane, + is HepG2 control, U is untreated, and LiCl is treated. Molecular weight markers sizes are shown to the left of the gel. For space concerns, Fig. 6A contains cropped images showing the change in Phospho-GSK3β levels.

An independent RNA-Seq analysis of PRH had identified increased expression of β-catenin and AKT1 at time points associated with an EMT^22^. As the β-catenin pathway is associated with EMT and is a modification often found in HCC^6^,^44^,^45^,^46^,^47^ we next attempted to determine what effect direct activation of β-catenin has on the glycosylation of PRH and examined them at a time point before the intrinsic EMT was observed (see Fig. 1). To that end, we used lithium chloride (LiCl) to activate this pathway in PRH and examined the glycosylation at 24 and 48 hours after treatment. Exposure of cells to LiCl leads to the phosphorylation and inactivation of glycogen synthase kinase 3β (GSK3β), thus preventing GSK3β-mediated phosphorylation and destabilization of β-catenin and increasing β-catenin signaling^48^. We first confirmed that LiCl treatment increases levels of the inactivating phosphorylation of GSK3β at serine-9 in PRH. As expected, immunoblotting of proteins isolated from PRH after 24 or 48 hours of treatment with LiCl showed elevated phosphorylation of GSK3β at serine-9 with no overall increase in GSK3β levels (Fig. 6A).

The N-linked glycan profile associated with lysates prepared from PRH either left untreated or treated with LiCl for 48 hours was analyzed (Fig. 6B–G). The released N-linked glycans were labeled with a fluorescent dye (2AB) and analyzed by normal phase HPLC as before. Samples were subsequently digested by sequential exoglycosidase treatment to identify glycan structures present in each peak as described in Fig. 2. At least two peaks indicate altered glycans in cells treated with LiCl as compared to the untreated controls (NT), and these have been identified as a di-sialylated biantennary glycan (GU 8.83) and a core fucosylated bi-antennary glycan (GU 7.55). While these changes are subtle, they have been consistently observed with independent batches of PRH (n = 4). In addition, all treatments and analysis (for each batch) were performed in duplicate. The level of the di-sialylated peak increased from 18% (±1.6) in the untreated control to 26% (±2.9%) in the LiCl treated sample (Fig. 6H). The level of core fucosylation (as found on the biantennary glycan) increased from 0.6% (±0.2) to 2.6% (±0.8) (Fig. 6I). In both cases, the difference is statistically significant (p < 0.05).

Confirmation of the HPLC analysis of N-linked glycans was obtained by lectin blotting. Lysates from a separate set of PRH, treated as before, were resolved by SDS-PAGE and analyzed by lectin blotting (Fig. 6J,K). Sialylated glycoproteins were detected using the SNA lectin as in Fig. 2 and potential alteration in fucosylation was determined using the recombinant N224Q rAAL (see Methods) (Fig. 6K). In agreement with the HPLC glycan analysis, increased levels of sialylation and fucosylation can be observed via lectin blotting following activation of the β-catenin pathway.

### Activation of the Akt signaling pathway or elevation of HIF1-α also leads to alterations in protein glycosylation

As activation of the β-catenin pathway lead to increases in core fucosylation and sialylation, we next examined whether changes in glycosylation could be observed by exposing PRH to conditions that activate additional cancer-associated signaling pathways. One pathway of interest was the AKT pathway, as the AKT1 gene was also up regulated in PRH as a function of time in culture^22^. Thus, in experiments similar to those described above, PRH were transfected with pMyrAkt1 or pMyrAkt2, which express myristolated forms of Akt 1 and 2 (see Methods), leading to constitutive Akt activation. In addition, PRH were treated with 1 mM dimethyloxalylglycine (DMOG) to elevate hypoxia inducible factor 1 (HIF-1α) expression, which has also been shown to induce an EMT in hepatocytes^49^.

**Figure 7:**
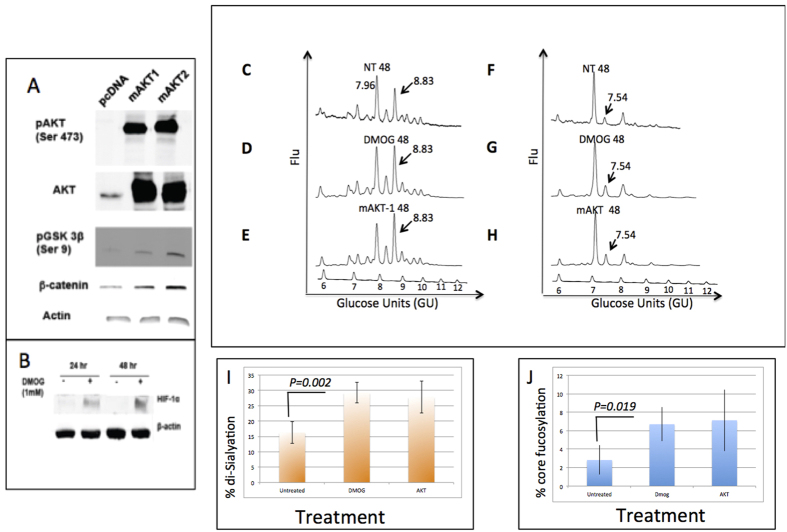
Changes in N-linked glycosylation can be observed with activation of AKT and HIF-1 α. PRH were transfected with a control plasmid (pcDNA) or with pMyrAkt1 or pMyrAkt2, to express myristolated Akt1 or 2. 48 hours post-transfection, levels of total Akt, phospho-Akt, phospho-GSK3β, and β-catenin were assessed by immunoblotting. (**B**) Cells were left untreated or treated for 24 or 48 hours with 1 mM dimethyloxalylglycine (DMOG) to elevate HIF1α. Cell lysates were analyzed by immunoblotting to detect HIF1α. In panels A and B, Actin is detected as a loading control. (**C–H**) Glycans were released from proteins from PRH left untreated (NT-48), treated with DMOG for 48 hrs (DMOG-48), or transfected with pMyrAkt1 for 48 hours and analyzed by normal phase HPLC as described. (**C–E**) N-linked glycan profiles from untreated, DMOG treated, or pMyrAkt1 transfected cells. (**F–H**) Samples were further digested with *Arthrobacter ureafaciens* sialidase. A peak showing increased levels of core fucosylation (GU 7.55) in DMOG treated cells and pMyrAkt1 transfected cells is indicated by an arrow. For panels C–H, glucose values are provided for the major peaks and the GU ladder is provided along the X-axis. Y-axis for these panels represents the fluorescent intensity of glycan. (**I,J**) Quantification of the alteration in sialylation (**I**) and fucosylation (**J**) following DMOG treatment or AKT induction. For space concerns, Fig. 7A contains cropped images.

Phosphorylated Ser 9-GSK-3β and increased levels of β-catenin were associated with myrAkt1 and 2 expression (Fig. 7A) and exposure of PRH to DMOG caused the expected elevation of HIF1α (Fig. 7B). Phosphorylation of Akt at Ser 473, an indicator of active Akt, was detected with an Akt-phospho-Ser473-specific antibody. Overall, these studies confirm that our treatments effectively regulate cancer-associated cellular signal-transduction pathways as intended.

The changes in N-linked glycosylation observed in the DMOG-treated PRH and the myrAkt1 and 2 expressing PRH were similar to those observed in the LiCl-treated cells. Increased levels of sialylation, in the form of a di-sialylated bi-antennary glycan, were apparent after 48 hours of exposure to DMOG (Panel D, GU 8.83) or 48 hours after transfection with pMyrAkt1 (Panel E, GU 8.83). Activation of the β-catenin pathway increased the level of the di-sialylated peak from 16% (±3.6) in the untreated control cells to 29% (±3.3%) in the DMOG-treated sample and 28% (±5.1) in the myrAkt1-expressing sample (Fig. 7I). These differences are statistically significant (p < 0.05). The level of bi-antennary core fucosylation (Fig. 7F–H, GU 7.54) increased from 3.3% (±1.7) in the untreated sample to 6.7% (±1.8) in the DMOG-treated sample and 7.1% (±3.4%) in the myrAkt1-expressing sample (Fig. 7J). Again, these differences are statistically significant (p < 0.05). Increases in core fucosylation could be prevented by use of the Akt inhibitor LY294002 suggesting a key role for Akt in the production of core fucosylated glycan (data not shown).

These results indicate that activation of specific cancer-associated pathways in PRH leads to changes in fucosylation and sialyation that are similar to those observed in human HCC samples^6^, supporting the use of PRH to model specific, known pathways in human HCC progression.

## Discussion

Alterations in N-linked glycosylation have been associated with the development of cancer. One specific modification, core fucosylation, has been observed in non-small cell lung carcinoma (NSCLC), ovarian cancer, and colon cancer and is used clinically in the diagnosis of hepatocellular carcinoma (HCC) in the form of the AFP-L3 assay^50^,^51^,^52^,^53^. However, why core fucosylation is increased in many of these conditions is unclear. In this study, we show that increased core fucosylation was directly associated with de-differentiation of hepatocytes and the appearance of markers of a transition of cells from an epithelial to a mesenchymal state. This finding is similar to that previously reported for NSCLC where increases were observed in the expression fucosyltransferase 8 (*FUT-8*), the enzyme that mediates core fucosylation^51^. Additionally, recent reports have indicated that both sialylation and fucosylation are involved in EMT^54^,^55^ and in liver regeneration^56^. In the case of sialylation, it was shown alpha 2,6 linked sialylation is increased dramatically following TGF-Beta -induced EMT. In addition, the inhibition of alpha 2,6 linked sialylation inhibited EMT, while the induction of alpha 2,6 linked sialylation promoted EMT^54^. Previous work has also shown that core fucosylation is required for proper TGF receptor activation as mice that lack FUT-8 have a severe dysreguation of TFG signalling^57^. Furthermore, a recent study showed that mice up-regulated FUT-8 following a partial hepatectomy and importantly, mice that lack FUT-8 were unable to regenerate the liver following a partial hepatectomy^56^. Our results are consistent with this and suggest a model where fucosylation is increased, and most likely required for a proper EMT.

In addition, we show that the activation of specific cancer-associated cellular signaling pathways can increase core fucosylation and induce additional glycoform alterations on hepatocyte proteins. Specifically, we were able to determine that increased levels of protein sialylation and α-1,6 linked core fucosylation are observed following activation of the β-catenin pathway. Activation of the Akt pathway or elevation of HIF-1α also resulted in increased levels of fucosylation and to a greater extent, sialylation. The singular activation of these cancer associated pathways lead to more moderate changes in glycosylation as compared to the intrinsic EMT but are consistent with what others have observed following activation of individual cancer pathways^58^.

The core fucosylated form of AFP, referred to as AFP-L3, has been used for a number of years as a prognostic biomarker of HCC. AFP-L3 has been shown to be associated with more aggressive disease with worse outcome^50^,^59^,^60^,^61^,^62^. Our observation that core fucosylation is associated with hepatocyte de-differentiation may explain this correlation, since it is well accepted that less differentiated cancers are more aggressive and associated with poor outcome^44^. Thus, if core fucosylation is a marker of de-differentiation, it could be used as a marker of a specific tumor type and have clinical value.

In conclusion, we show that increased core fucosylation, a modification of N-linked glycoproteins associated with HCC, is associated with the process of cellular de-differentiation in PRH. We believe that this knowledge will help to use this glycan modification in a clinically meaningful way.

## Methods

### Isolation and culture of primary rat hepatocytes

Surgery and isolation of hepatocytes from rats were approved by the Institutional Animal Care and Use Committee of the Drexel University College of Medicine (Protocol # 20057) and complied with the Animal Welfare Act, the Public Health Service Policy on Humane Care and Use of Laboratory Animals, and the NIH Guide for the Care and Use of Laboratory Animals (2011). Primary rat hepatocytes (PRH) were isolated using a 2-step perfusion method as previously described^20^. The PRH were plated on collagen-coated tissue culture plates at approximately 2.0 × 10^6^ cells per 60 mm plate (~80% confluent) in Williams E medium supplemented with 2.0 mM L-glutamine, 1.0 mM sodium pyruvate, 4.0 μg/ml Insulin/Transferrin/Selenium (ITS), 5.0 μg/ml hydrocortisone, 5.0 ng/ml epidermal growth factor (EGF), 10 μg/ml gentamycin, and 2% dimethyl sulfoxide (DMSO) and maintained at 37°C in 5% CO_2_ as previously described^21^. Animal surgery and hepatocyte isolation complied with all relevant federal and institutional policies. For all data shown, experimental and statistical analyses were performed on independent PRH batches (n = 4).

### N-linked glycan analysis

Cells were lysed in 0.5 X Radioimmunoprecipitation assay buffer (RIPA; Thermo Scientific) using procedures reported elsewhere^63^. 250 μg of protein was brought to a volume 45 μl (in 1X PBS) and boiled for five minutes. After cooling, 2.5 μl of NP-40 (10%) was added followed by the addition of 30 U PNGase F PRIME (Bulldog Bio, Portmouth, NH) and incubated overnight at 37 °C. Glycans were purified using HyperSep Hypercarb porous graphite carbon columns (Thermo Fisher Scientific Inc., Philadelphia, PA). Eluted glycans were fluorescently labeled using 2-aminobenzamide using methods previously reported^31^. The glycans were further purified with paper chromatography and filtered using a 0.22 μm syringe filter. Fluorescently labeled glycans were subsequently analyzed using the Waters Alliance high-performance liquid chromatography system with a normal phase column (TSK amide 80 columns) complemented with a Waters fluorescence detector and quantified using the Millennium Chromatography Manager (Waters Corporation, Milford, MA) as has been done previously by us and others. Further description of the methods, column diameter, length, packing and particle size of column can be found elsewhere^6^,^8^,^30^,^31^,^32^,^33^,^37^,^38^,^41^,^64^,^65^,^66^,^67^. Glycan structures were identified by calculating the glucose unit value and exoglycosidase digestion, as described previously^14^,^68^.

### Immunoblotting

20 μg of protein lysate from PRH were resolved SDS-PAGE on 4–20% polyacrylamide gradient gels, transferred to polyvinylidene difluoride (Millipore) membranes, and blocked with 5% powdered milk in 0.1% Tween20-PBS at room temperature for one hour. After incubation with primary antibody at a 1 to 1,000 dilution in LI-COR blocking buffer (Cat #927-40000) for 2 hours at room temperature, the blot was washed three times with 0.1% Tween20-PBS for 10 minutes/each wash. All antibodies (rabbit polyclonal N-cadherin (ab18203), vimentin (ab92547), caveolin-1 (ab18199), and alpha smooth muscle Actin antibody (ab5694)) were from Abcam (Cambridge, MA). The membrane was incubated with secondary antibody (IR-800 conjugated donkey anti-rabbit IgG from LI-COR, Lincoln Nebraska) at a 1 to 10,000 dilution in Carbo-Free blocking buffer (Vector Labs, Burlington, CA, Cat #Sp-5040) for 1 hour at room temperature. Blots were washed 3 times with 0.1% Tween20-PBS and imaged using the Odyssey^®^ Infrared Imaging System (LI-COR). In the case of lectin blotting, fucosylation was detected using a recombinant N224Q rAAL lectin. This is a recombinant AAL lectin that has been modified to increase binding to core fucosylated glycan^42^. For sialic acid detection, we used commercially purchased Sambucus nigra lectin (SNA, Vector Labs, Cat #B-1305), which binds preferentially to sialic acid attached to terminal galactose in α-2,6 and to a lesser degree, α-2,3 linkage. In all cases, biotinylated lectin was used and bound lectin detected using streptavidin conjugated IR800 dye.

### Reverse transcriptase PCR of primary rat hepatocyte genes

Briefly, PRH were grown for the indicated length of time and the total RN was extracted from primary rat hepatocytes using Trizol reagent (Invitrogen). Two fold dilutions of the RNA was performed with respective primer pairs to determine the minimum amount of RNA necessary for amplification (Supplementary Figure/Data not shown). Based on this estimate, 250 ng of RNA was reverse transcribed using reagents from the One step RT-PCR kit (Qiagen) in a final volume of 20ul. Samples were assayed in triplicates and the obtained results were normalized against control 18SrRNA. The primer pairs used were designed using primer blast and were as follows: 18S ribosomal RNA: Forward (F), AAA CGG CTA CCA CAT CCA AG; Reverse (R), GAG CTG GAA TTA CCG CGG CT. Rat Alpha (1,6) fucosyltransferase, F-ACG CAC AGA CAA AGT GGG AA; R-TCA CGC CCC GAA GTG AAT TT. Rat GDP-mannose 4, 6-dehydratase (Gmds), F-AGC CGG TCA GTA GCT AAG A; R-CTA TGA CAA AGT CCT CCG GC. Rat GDP-fucosetransporter (Slc35c1), F-GGT GAT CTG ATC CCT GCC G; R-GGG AGA AGG GGC CTA ACT AA. Rat GDP-fucosetransporter (Slc35c2), F-TCC TTC TCC ACA GCA CAA TG; R-AAC TGG TAG TCT CCA GAA GAT GG. Rat GDP-4-keto-6- deoxy-mannose-3, 5-epimerase-4-reductase (Tsta1), F-CAT CGC AGG TGA GAC TTA CA; R-TGC ACG TTC TTC CTC CAG AA. Rat GDP- fucose pyrophosphorylase (Fpgt), F-TAC AGT CAA CGC CTT CCC AA; R-AGG CTG GTC AAA GGC AAT GT.

### Tissue micro array and lectin histochemistry

Formalin-fixed paraffin embedded tissue microarray (FFPE-TMA) slides (Catalog No.: Z7020059, Lot No.: B506168) were purchased from Biochain, Inc. (Newark, CA). This array consisted of 16 cases of liver cancer, each in duplicate, with corresponding uninvolved tissue from the same patient acting as controls. All the tissues were from surgical resection. Patients had a mean age of 47.56 years (range of 33–68 years) with an 8:1 ratio of males to females. Samples were from patients with multiple etiologies of liver disease and were classified using the TNM classification system. Briefly, T1 = Solitary tumor without vascular invasion; T2 = Solitary tumor with vascular invasion or multiple tumors none larger than 5 cm; T3 - Multiple tumors more than 5 cm, or tumor involving a major branch of the portal or hepatic vein(s); T4 - Tumor(s) with direct invasion of adjacent organs other than the gallbladder or with perforation of visceral peritoneum. Tissue slides were deparaffinized by using PROTOCOL SafeClear II clearing agent (Fisher Scientific, Pittsburg, PA), followed by rehydration though a series of graded ethanol. Lectin histochemistry was performed at room temperature, unless otherwise indicated. Endogenous peroxidase activity was blocked using 3% hydrogen peroxide (H_2_O_2_), followed by heat-mediated antigen retrieval using Universal Antigen Retrieval Reagent (R&D Systems, Minneapolis, MN). Tissues were fixed with 4% formaldehyde solution followed by permeabilization with 0.5% IGEPAL. Prior to staining, FFPE-TMAs were blocked using serum-free Dako Protein Block (Carpinteria, CA). Following the blocking step, the detection of fucosylation was conducted using biotinylated recombinant N224Q rAAL lectin, diluted in Dako High Background Reducing Diluent solution (Carpinteria, CA) to make the working final concentration of 500 ng/mL. Bound, biotinylated lectin was detected using streptavidin horseradish peroxidase (Vector Laboratory, Burlingame, CA), and color was developed using 3,3′-diaminobenzidine (DAB) Chromogen (Dako, Carpinteria, CA). TMAs were counterstained with Harris-modified hematoxylin (Sigma Aldrich, St. Louis, MO). Slides were visualized using an E200 Binocular Compound Biological Microscope from Nikon Instrument (Melville, NY) and an IX71 Inverted Microscope from Olympus Imaging America, Inc. (Center Valley, PA).

### Matrigel overlay

PRH were plated in 60 mm plates as described above. One set of cells was overlaid with Matrigel (BD Biosciences) 60 hours after plating as described previously^69^. Briefly, Matrigel was diluted 1:3 in the PRH growth medium, and 250 μl of the diluted Matrigel was overlaid on the culture dishes that contained 750 μl of the growth medium. For the control set of primary hepatocytes, the medium was changed at a time coinciding with the Matrigel overlay. The Matrigel-overlaid cells were collected by washing 3 times with cold PBS, followed by incubation with 200 μl of Dispase (Corning^®^ Matrigel^®^™ Basement Membrane Matrix Dispase) at 37 °C. Subsequently, the cells removed and spun down at 2000 rpm for 5 min. The supernatant was removed and the cells were washed with PBS and spun down again at 2000 rpm for 5 min. The washing step was repeated three times and the cell pellet was lysed in 0.5 X RIPA before as described above. We have attempted to perform N-linked glycan analysis on the last wash step of the cells but no glycan could be detected (data not shown).

### Activation of cancer-associated signal transduction pathways in PRH

β-catenin was activated by LiCl-mediated inhibition of GSK-3β^51^ immediately after plating. Activation of the Wnt/β-catenin pathway was determined by assessing accumulation of β-catenin in treated cells, an indicator of active β-catenin. The hepatocytes were transfected 24 hours after plating with plasmids expressing forms of Akt1 and Akt2 that become myristoylated, which targets these proteins to the plasma membrane and causes their constitutive activation^70^. PRH were transfected with Lipofectamine 2000, according to the manufacturer’s instructions. These plasmids were constructed by William Sellers and purchased from Addgene (Cambridge, MA). The plasmid pMyrAkt1 (Addgene plasmid #9008) was used to generate myrAkt1 and the plasmid pMyrAkt2 (Addgene plasmid #9016) was used to generate myrAkt2. Cells transfected with pMyrAkt1 or pMyrAkt2 were collected at specific time points after transfection by washing with ice cold phosphate-buffered saline (PBS) followed by scraping the cells in PBS. The cells were collected by centrifugation and then used for immunoblot analysis or fucosylation studies. For immunoblot analysis, the cell pellet was lysed in 0.8% sodium dodecyl sulfate (SDS) buffer (0.8% SDS, 240 mM Tris (pH6.8), 10% glycerol), total protein was quantified, and equal amounts of total protein were loaded for analysis by SDS polyacrylamide gel electrophoresis (PAGE). Phospho-Akt, Akt, β-catenin, and phospho-GSK3β antibodies were purchased from Cell Signaling Technology, Inc. (Boston, MA); the β-actin antibody was purchased from Sigma-Aldrich (St. Louis, MO).

For induction of hypoxia inducible factor 1α (HIF-1α), cells were exposed to 1 mM of the prolyl-4-hydroxylase inhibitor, dimethyloxalylglycine (DMOG), which elevates HIF-1α, mimicking effects associated with hypoxia^71^. Treatment with 1 mM DMOG was started 24 hours after plating. Cells were treated with DMOG for the indicated time periods and then collected. Cells were washed with ice-cold PBS and collected by centrifugation. Cell pellets were used for fucosylation studies or for immunoblot analysis as described above; the anti-HIF-1α was purchased from Santa Cruz Biotechnology (Santa Cruz Biotechnology, Dallas, TX).

### Statistics

In all relevant cases, results are given as the mean with the standard deviation from the mean. Comparisons between times and treatments were carried out using 2-tailed Mann-Whitney tests. A *p* value of less than 0.05 was considered significant. All data were analyzed using Graphpad Prism 5.0 (GraphPad Software Inc., La Jolla, CA).

## Additional Information

**How to cite this article**: Mehta, A. *et al*. Intrinsic hepatocyte dedifferentiation is accompanied by upregulation of mesenchymal markers, protein sialylation and core alpha 1,6 linked fucosylation. *Sci. Rep.* **6**, 27965; doi: 10.1038/srep27965 (2016).

## Supplementary Material

Supplementary Information

## Figures and Tables

**Table 1:** Lectin staining of tissue micro-array with the N224Q AAL lectin.

**HCC Sample**^1^	**Grade (I–IV)**^2^	**Stage (1, 2, 3, 4)**^3^	**Age**^4^ **(years)**	**Gender**^5^	**Level of hepatocyte staining**^6^
1	I-II	2	52	M	+
2	I-II	2	40	M	+
3	I-II	2	61	M	+
4	I-II	3	43	M	+
5	II	2	40	M	++
6	II	2	39	M	+
7	II	2	47	M	+
8	II	1	52	F	+++
9	II	2	41	M	+
10	II	3	41	M	+
11	II	3	57	M	+
12	II-III	2	36	M	++
13	II-III	2	46	F	++
14	III	2	33	M	++++
15	III	2	65	M	+
16	III	2	68	M	++++
Adjacent normal tissue^7^	NA	NA	47.56 (SD: 10.56)	NA	+

^1^HCC samples from 16 patients were provided in duplicate on the tissue microarray. Each individual with HCC also had adjacent normal tissue on the array. Thus, a total of 48 samples from 16 individuals were provided.

^2^The grade of tumor was provided for each sample. Grade I, well differentiated tissue; Grade II, moderately differentiated tissue; Grade III, poorly differentiated tissue; Grade IV, un-differentiated tissue.

^3^HCC was classified using the TNM classification system. Briefly, T1 = Solitary tumor without vascular invasion; T2 = Solitary tumor with vascular invasion or multiple tumors none larger than 5 cm; T3 - Multiple tumors more than 5 cm, or tumor involving a major branch of the portal or hepatic vein(s); T4 - Tumor(s) with direct invasion of adjacent organs other than the gallbladder or with perforation of visceral peritoneum.

^4^Age of individual.

^5^Gender of sample. M, Male; F, female.

^6^Level of tissue staining with the N224Q rAAL lectin, which has enhanced staining of core fucosylated glycan (see Methods). The level of staining was qualitative as determined by blinded analysis of samples and judgment of staining from: no staining (−); mild staining of hepatocytes (+); moderate staining of hepatocytes (++); moderate to strong staining of hepatocytes (+++); very strong staining of hepatocytes (++++). All staining was performed in duplicate and compared with the level of hepatocyte staining observed in the matching adjacent normal tissue. Data for each tissue spot is shown in Supplementary Figures S2 and S3.

^7^Each HCC sample had an adjacent normal tissue provided. Staining was observed in the sinusoids. The mean age of all patients with the standard deviation are provided.
