# Supplementary material for: Intrinsic hepatocyte dedifferentiation is accompanied by upregulation of mesenchymal markers, protein sialylation and core alpha 1,6 linked fucosylation
Source: Sci Rep. 2016 Jun 22;6:27965. doi: 10.1038/srep27965 (PMC4916422; doi:10.1038/srep27965)
Supplement: Supplementary Information [file srep27965-s1.pdf]

Intrinsic hepatocyte dedifferentiation is accompanied by upregulation of mesenchymal markers, protein sialylation and core alpha 1,6 linked fucosylation*

Anand Mehta, Mary Ann Comunale, Siddhartha Rawat, Jessica C. Casciano, Jason Lamontagne, Harmin Herrera, Aarti Ramanathan, Lucy Betesh, Mengjun Wang, Pamela Norton, Laura F. Steel and Michael J. Bouchard

Supplementary Figure S1-S3

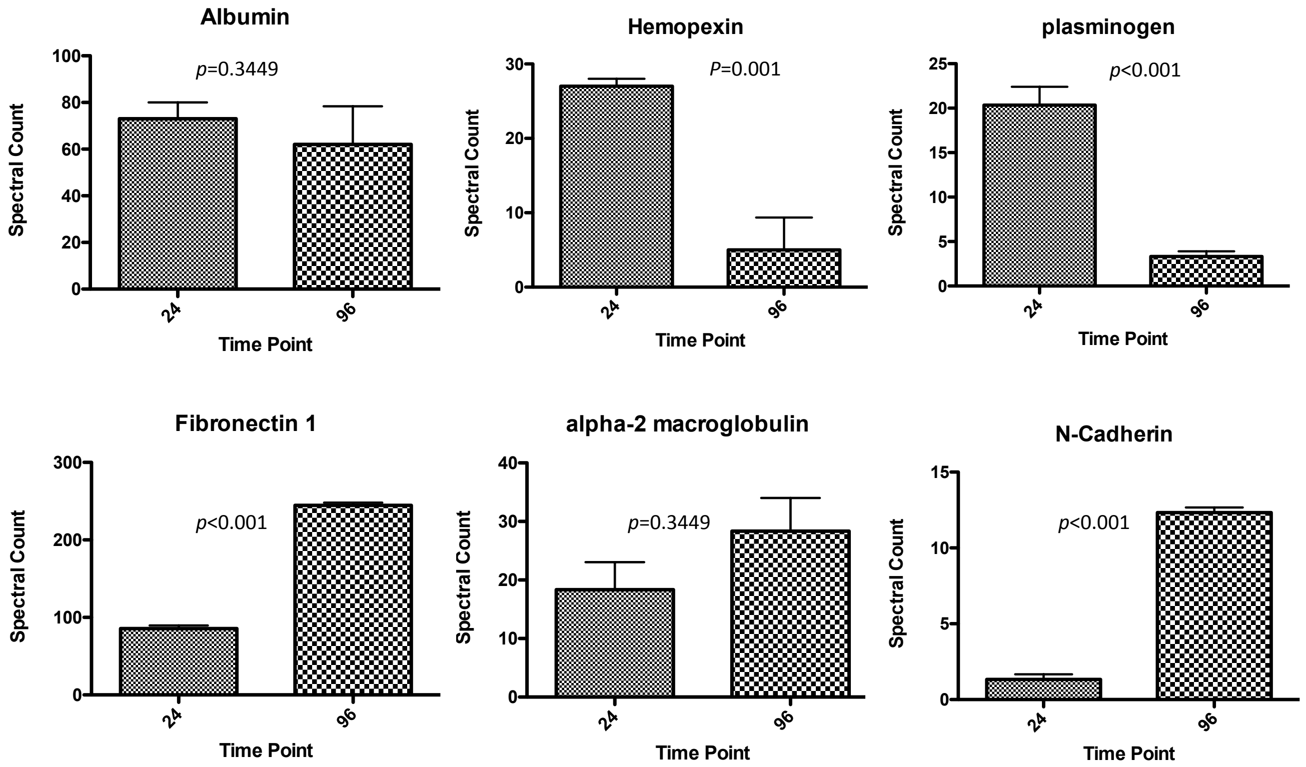

Supplementary Figure 1. Mass Spectrometry of cell associated protein material from PRH after 24 or 96 hours post culture. Analysis was done with triplicate samples at time point 24hours and 96hours. Mass spectrometry was completed on a Thermo LTQ-Orbitrap-Velos Pro ETD mass spectrometer. Separation was achieved using an Ultimate 3000 Rapid Separation nano LC system (Dionex, Sunnyvale, CA). As this figure shows, while the level of albumin is slightly altered, this was not dramatically reduced. In contrast, hemopexin levels and plasminogen levels, are reduced dramatically. In addition, proteins such as fibronectin and N-cadherin, which are indicative of an EMT are increased. The p value is given and $p<0.05$ was used to determine statistical significance. Increases in N-cadherin were also detected by immunoblotting in Figure 1E.

Supplementary Figure S2. Lectin immunohistochemistry analysis of tumor and tumor-adjacent tissue from five patients with Grade I-II (defined as well or moderately differentiated tissue) HCC. Rows A to C: Tissue microarrays were purchased and analyzed to detect core fucosylation as described in Methods (main text). Rows A and B are HCC samples from a single individual. Row C is adjacent normal tissue from the same individual. Both 4X and 20X resolution images are shown, as indicated. Rows D to F, G to I, J to L, M to O, P to R, S to U, V to X, Y to AA, BB to DD and EE to GG show similar tumor and adjacent tissue samples from four additional patients. Patient samples shown in V to X were considered positive. That is, the HCC tissue had greater staining of hepatocytes as compared to the control adjacent tissue. All other HCC samples were considered negative, That is, the HCC tissue had no greater staining of hepatocytes as compared to the control adjacent tissue.

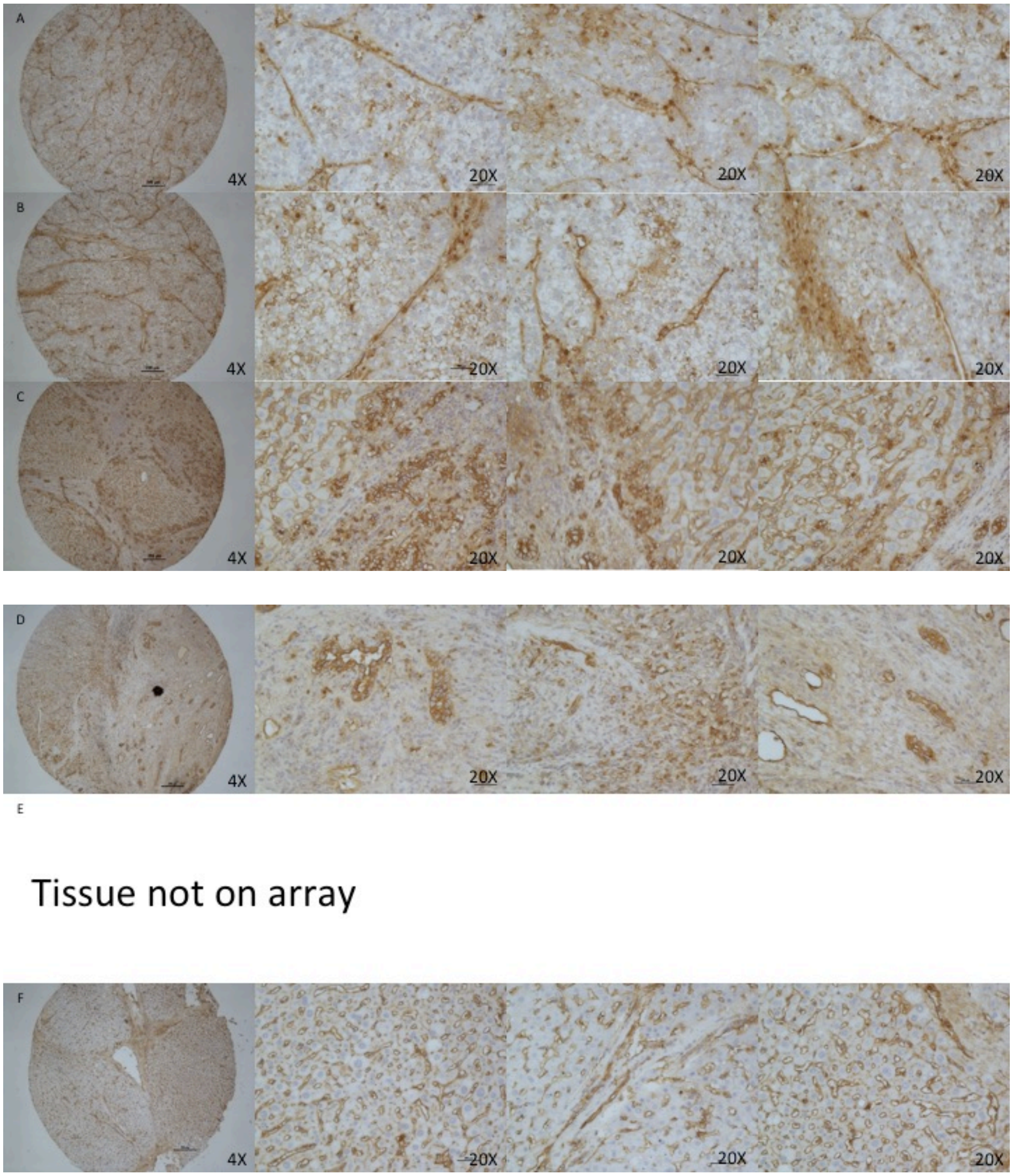

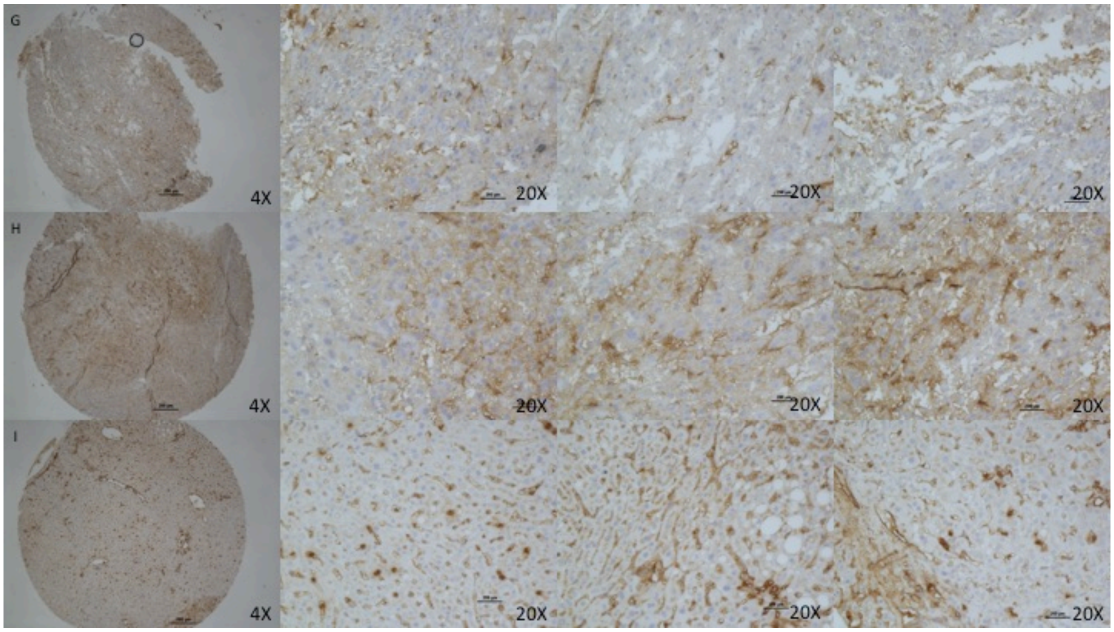
G
4X
20X
20X
20X
H
4X
20X
20X
20X
I
4X
20X
20X
20X

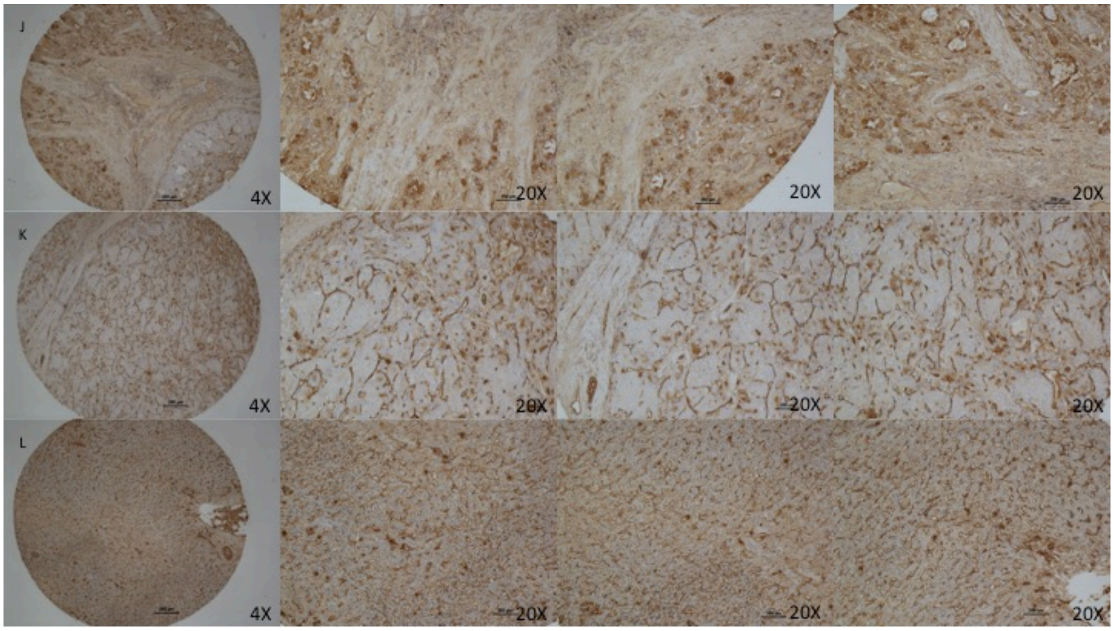
J
4X
20X
20X
20X
K
4X
20X
20X
20X
L
4X
20X
20X
20X

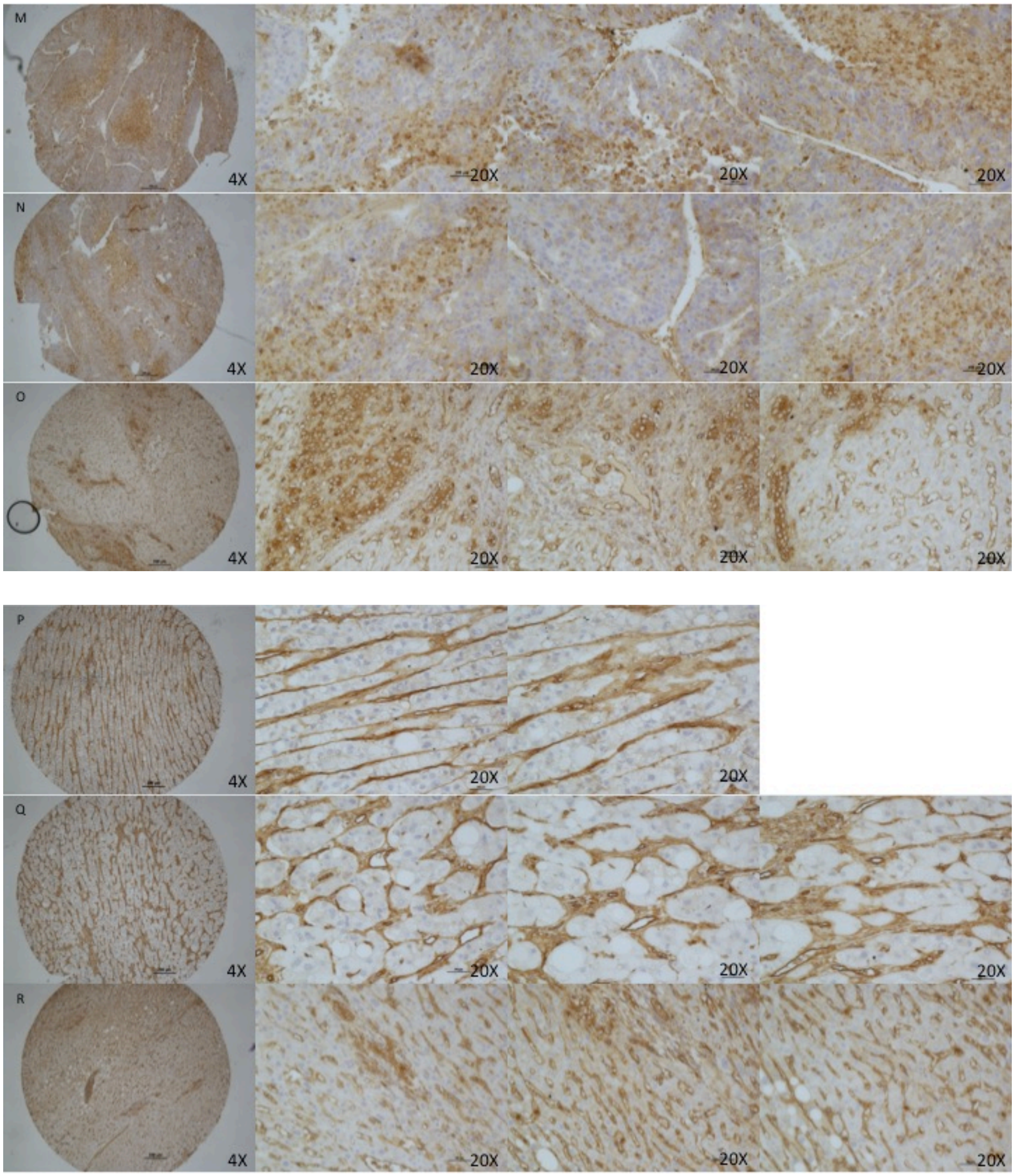
M
4X
20X
20X
20X
N
4X
20X
20X
20X
O
4X
20X
20X
20X
P
4X
20X
20X
Q
4X
20X
20X
20X
R
4X
20X
20X
20X

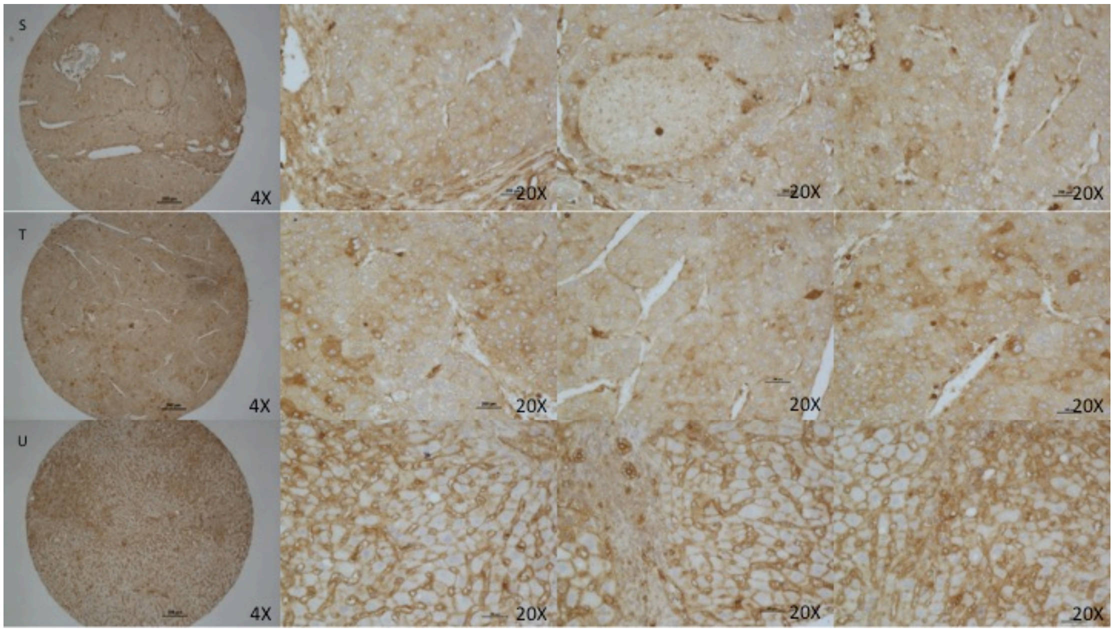
S
4X
20X
20X
20X
T
4X
20X
20X
20X
U
4X
20X
20X
20X

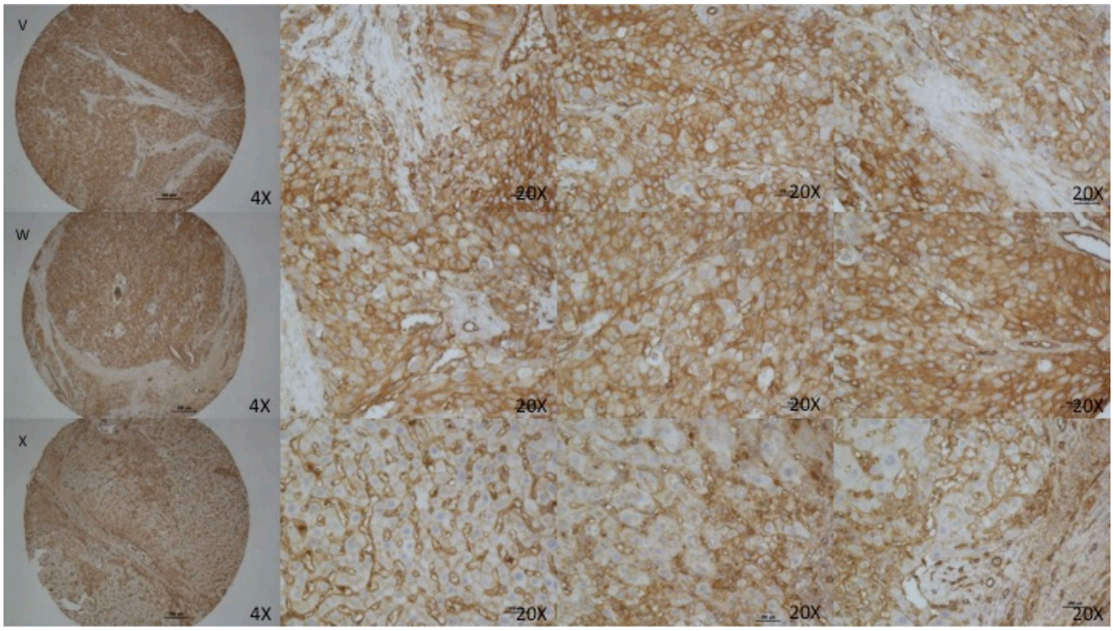
V
4X
20X
20X
20X
W
4X
20X
20X
20X
X
4X
20X
20X
20X

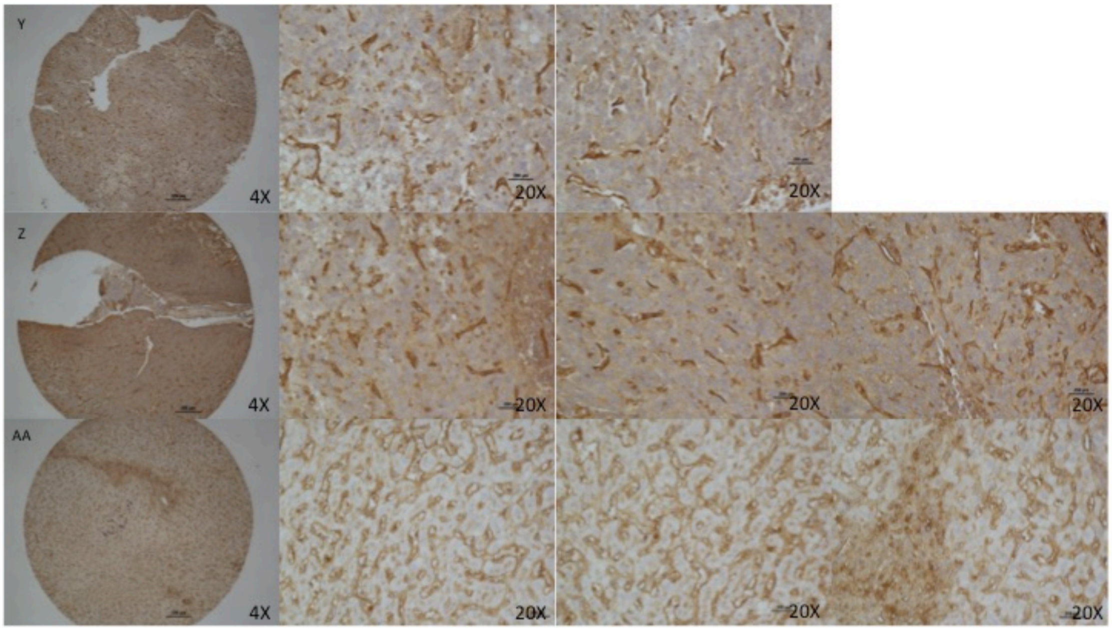
Y
4X
20X
20X
Z
4X
20X
20X
20X
AA
4X
20X
20X
20X

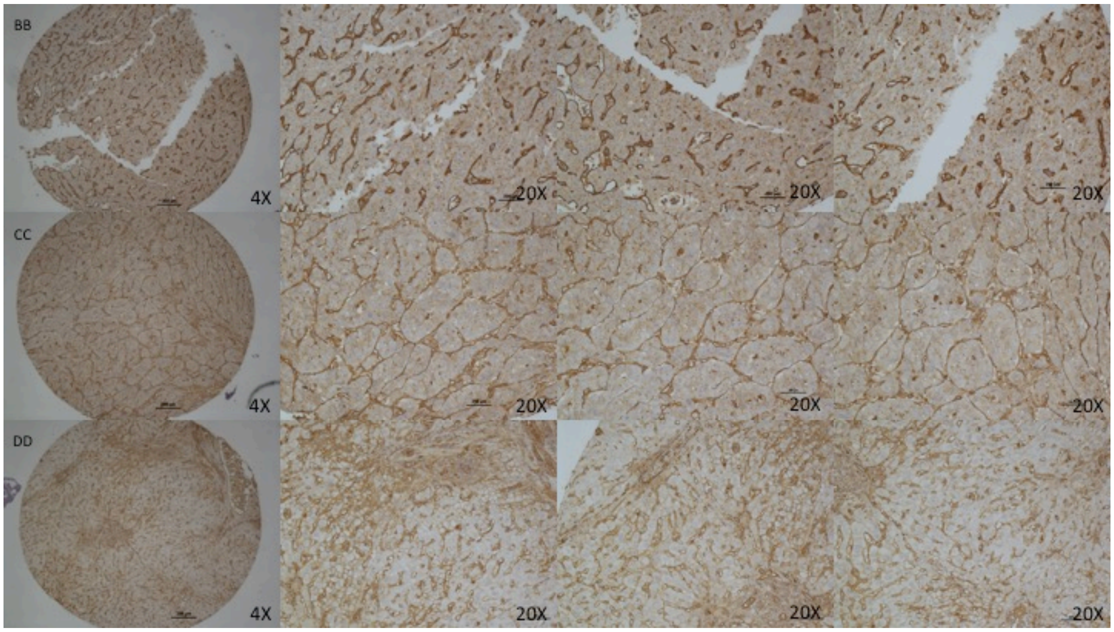
BB
4X
20X
20X
20X
CC
4X
20X
20X
20X
DD
4X
20X
20X
20X

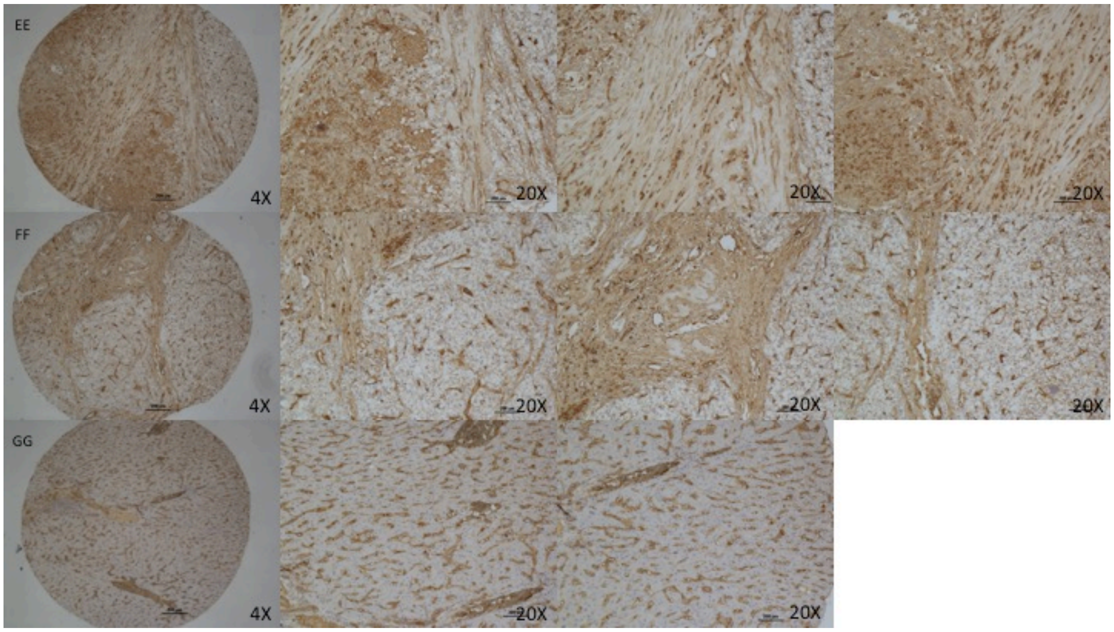
EE
4X
20X
20X
20X
FF
4X
20X
20X
20X
GG
4X
20X
20X

Supplementary Figure S3. Lectin immunohistochemistry analysis of tumor and tumor-adjacent tissue from five patients with Grade III (poorly differentiated) HCC. Tissue microarrays were purchased and analyzed to detect core fucosylation as described in Methods (main text). Rows A and B are HCC samples from a single individual. Row C is adjacent normal tissue from the same individual. Both 4X and 20X resolution images are shown, as indicated. Rows D to F, G to I, J to L, and M to O show similar tumor and adjacent tissue samples from four additional patients.

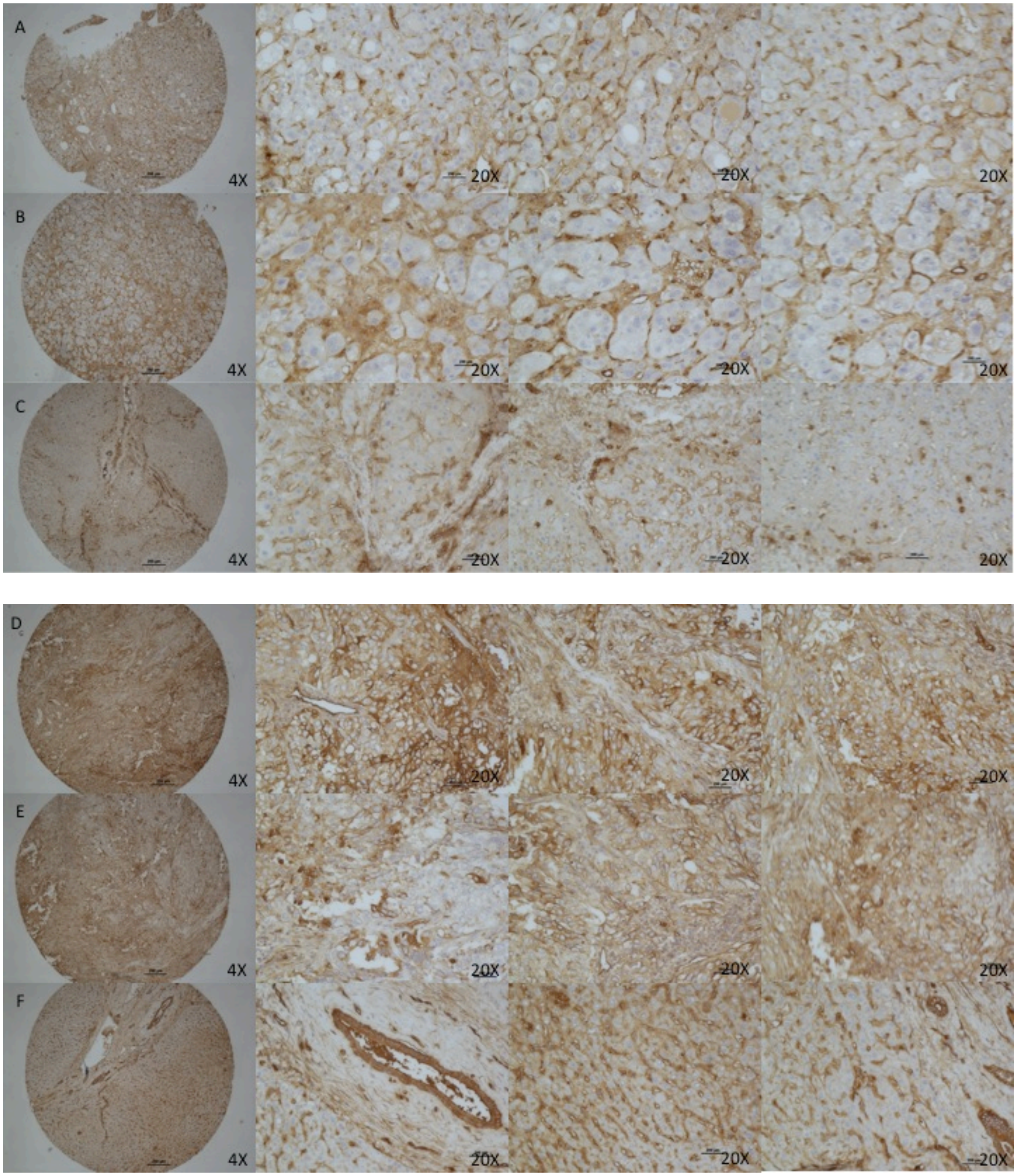

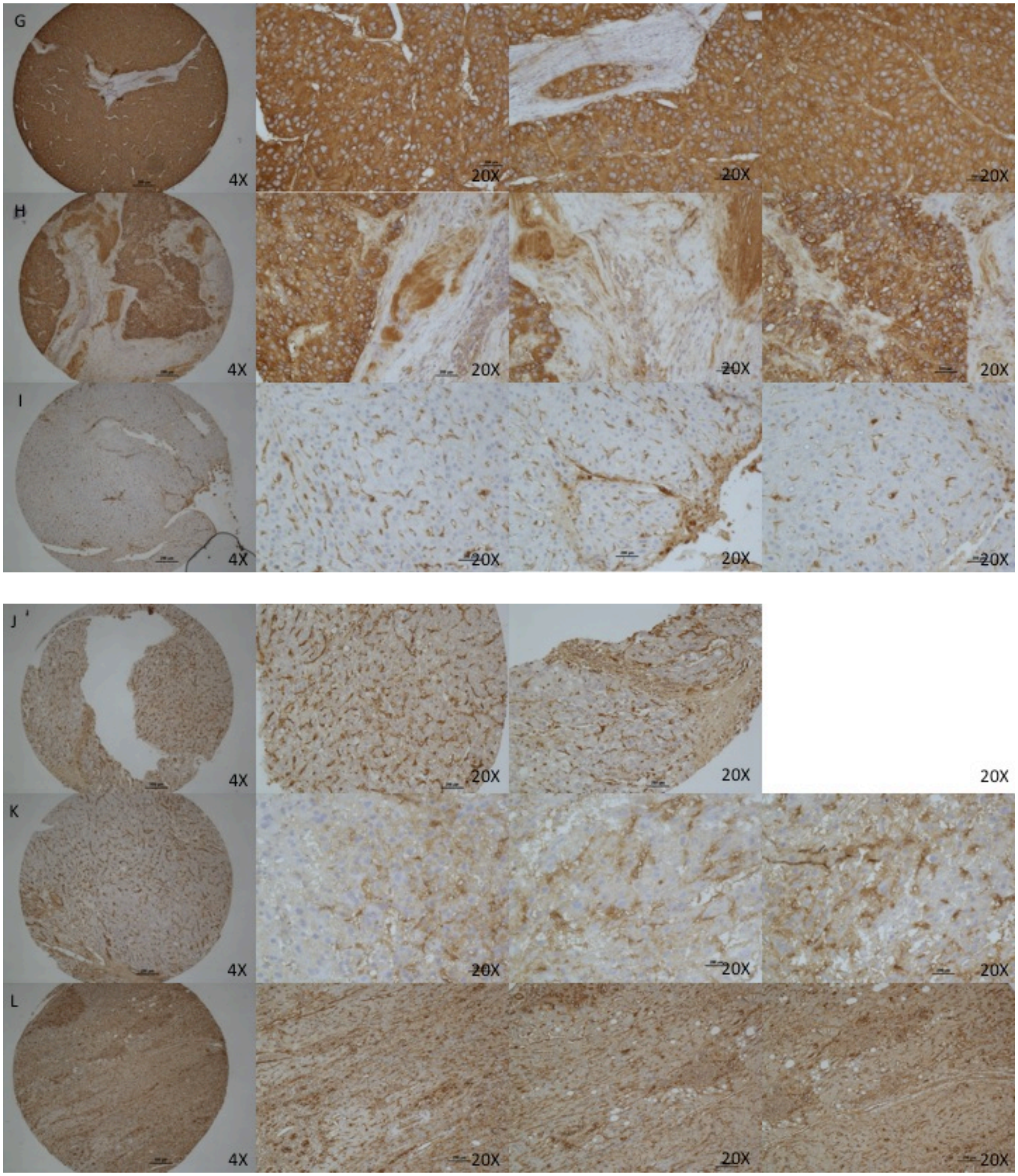
G
4X
20X
20X
20X
H
4X
20X
20X
20X
I
4X
20X
20X
20X
J
4X
20X
20X
20X
K
4X
20X
20X
20X
L
4X
20X
20X
20X

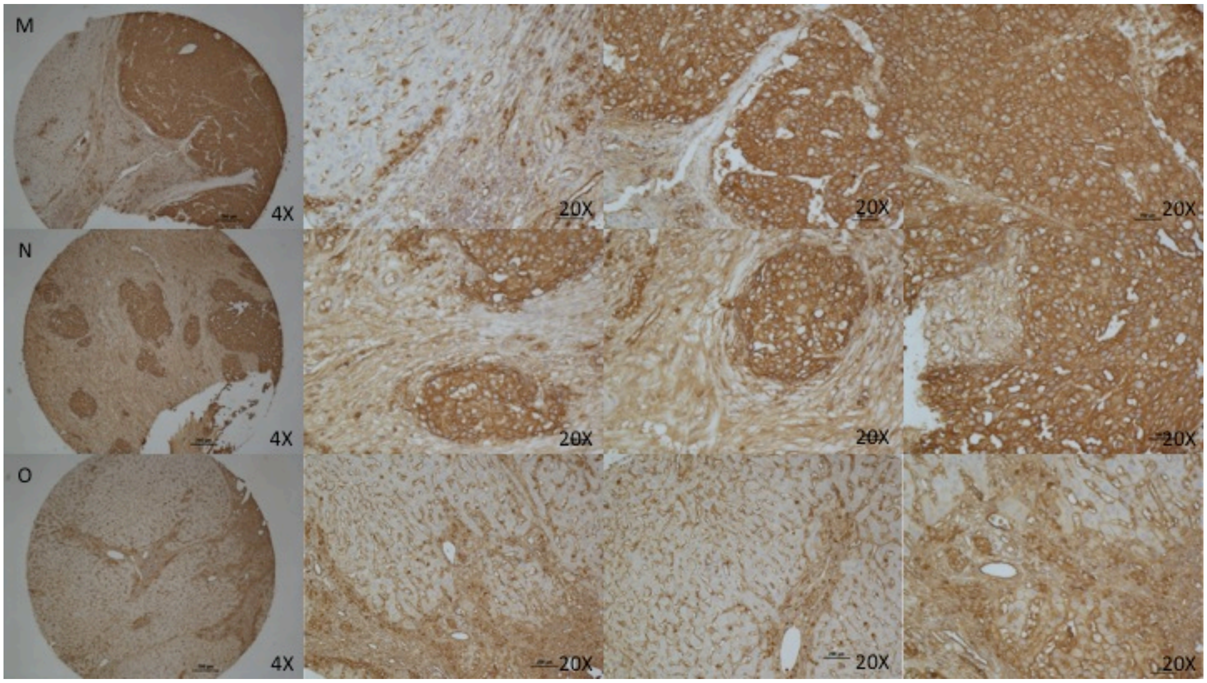
M
4X
20X
20X
20X
N
4X
20X
20X
20X
O
4X
20X
20X
20X
